# Oxygen consumption efficiency in firefighters: roles of fatigue and rescue task

**DOI:** 10.3389/fphys.2025.1708050

**Published:** 2025-11-26

**Authors:** Xinxin Zhang, Chengxiu Li, Yong Zhou, Weiguo Liu

**Affiliations:** 1 School of Physical Education, Shaanxi Normal University, Xi’an, China; 2 College of Physical Education and Health, Guangxi Normal University, Guilin, China; 3 College of Physical Education, University of South China, Hunan, China; 4 College of Physical Education and Health, Guilin Institute of Information Technology, Guilin, China

**Keywords:** firefighter, fatigue, rescue task, oxygen consumption, task load, rescue methods

## Abstract

**Objective:**

This study compared standardized oxygen consumption responses during rescue tasks with varying loads and methods across different fatigue states in firefighters to identify optimal rescue strategies and enhance operational efficiency.

**Methods:**

Sixty-three professional healthy male firefighters were recruited. Oxygen consumption was measured using a wearable metabolic system as they performed 5 × 20-m shuttle sprints with different task loads (10 kg, 20 kg, 30 kg) and rescue methods (shoulder-, cradle-, hand-carrying) under five fatigue states (non-fatigue, whole-body, and mild/moderate/severe knee fatigue).

**Results:**

A mixed-model ANOVA revealed a significant main effect of task load on standardized oxygen consumption (F = 620.61, P < 0.001, ${ƞ}_{p}^{2}$ = 0.798). Post-hoc tests showed that standardized oxygen consumption was significantly lower during medium-load (2.464 ± 0.616 mL/min/kg^2^) and large-load tasks (1.615 ± 0.317 mL/min/kg^2^) compared to small-load tasks (4.718 ± 1.043 mL/min/kg^2^) (all P < 0.05). Consumption during large-load tasks was also significantly lower than during medium-load tasks (P < 0.05). The main effects of fatigue states and rescue methods were not significant.

**Conclusion:**

Task load is the primary factor influencing oxygen efficiency during high-intensity shuttle sprints. Large-load tasks resulted in significantly lower standardized oxygen consumption across all fatigue states and rescue methods, indicating a “small load-low efficiency” phenomenon. Prioritizing large-load (30 kg) tasks in time-critical emergencies may improve rescue efficiency.

## Introduction

Given firefighters’ unique working environment and occupational demands, they are often required to perform rescue tasks under extreme conditions (Lavender et al., 2014). The complexity and intensity of these tasks pose substantial occupational safety risks (Bai et al., 2021; Ras et al., 2024; Zhang et al., 2024). Enhancing firefighters’ rescue performance has become a key research priority, as both their task execution efficiency and the extent to which they can leverage their professional skills directly affect operational outcomes (Fyock-Martin et al., 2020; Weidinger, 2022), with broader implications for national and public safety (Dowdall-Thomae et al., 2012; Heydari et al., 2022). Addressing how to simultaneously improve rescue efficiency while reducing safety hazards and injury risks constitutes a pressing scientific challenge in firefighting and rescue (Zhang et al., 2024; Kesler et al., 2018a).

Firefighting rescue tasks are characterized by two notable features (Zhang et al., 2024). First, fatigue during operations is virtually unavoidable due to the persistent state of readiness and inherently high-risk nature of rescue work (Tang et al., 2025). Critically, fatigue has been demonstrated to affect physiological efficiency in firefighters detrimentally. For instance, Kesler et al. (2018a) reported impaired ventilatory efficiency—manifested as a higher oxygen consumption for the same work rate—when firefighters performed occupational tasks in a fatigued state. This evidence underscores that fatigue limits performance and compromises metabolic economy, making optimizing rescue strategies under fatigue a crucial safety and efficiency concern. Second, the urgency of specific firefighting scenarios, particularly during critical life-saving interventions or emergency egress, can necessitate short bursts of high-speed movement to execute time-sensitive tasks, despite standard protocols advising against sustained running in hazardous environments (Zhang et al., 2024). This need is particularly acute in oxygen-constrained environments, where efficient oxygen utilization is crucial for task completion and firefighter safety (Zhang et al., 2024; von et al., 2006; Perroni et al., 2015). Oxygen consumption levels during rescue tasks thus serve as a critical metric for evaluating task efficiency and firefighter safety (Von et al., 2006). Nevertheless, existing studies have predominantly emphasized firefighting equipment (Kesler et al., 2018b; Kesler et al., 2018c; Bustos et al., 2021; Deng et al., 2018; Li et al., 2023; Morel et al., 2014; Rezazadeh and Torvi, 2011), strength and conditioning (Park et al., 2018; Adetona et al., 2016; Cuenca-Lozano and Ramirez-Garcia, 2023; Igboanugo et al., 2021; McMorrow and Feairheller, 2022; Lentz et al., 2019), and injury prevention (Gallanter and Bozeman, 2002; Rabbitts et al., 2004; Carr-Pries et al., 2022), with limited research on optimizing rescue strategies from the perspective of oxygen consumption. In particular, experimental investigations into task-specific and method-specific rescue strategies remain scarce. In practice, firefighters must make rapid decisions regarding which rescue method to employ under specific task loads and fatigue states to maximize efficiency while minimizing safety risks (Zhang et al., 2024).

To address this gap, the present study employed a three-factor mixed experimental design, incorporating 5 fatigue states (non-fatigue, whole-body fatigue, mild knee fatigue, moderate knee fatigue, and severe knee fatigue) × 3 task loads (10 kg small, 20 kg medium, and 30 kg large) × 3 rescue methods (shoulder-carrying, cradle-carrying, and hand-carrying). This study aimed to compare standardized oxygen consumption responses across different fatigue states and rescue conditions among professional firefighters, focusing on task load and rescue method, to identify task configurations that optimize oxygen utilization efficiency. Therefore, the following experimental design was constructed to test these relationships directly by modeling key firefighting task demands.

## Methods

### Experimental design

In light of the dual characteristics of firefighting and rescue tasks—namely, their high susceptibility to fatigue and the necessity of running-based task execution (Zhang et al., 2024)—a fatigue induction protocol based on running to exhaustion was developed to model both non-fatigued and whole-body fatigued states. Given the high vulnerability of firefighters’ lower limbs and the principle of rescue initiative (Perroni et al., 2015; Wang and Wang, 2022), the quadriceps femoris, the primary muscle group of the knee joint, was chosen as the target for localized fatigue induction. Three distinct knee fatigue models were established: mild fatigue (25% reduction in peak knee moment), moderate fatigue (50% reduction), and severe fatigue (75% reduction) (Gear, 2011).

Task load design was informed by typical firefighting equipment weights (Tang et al., 2025): large load (30 kg, approximating the weight of a fire ladder), medium load (20 kg, based on a gas cylinder), and small load (10 kg, based on a fire hose). In parallel, three common rescue methods were adopted according to standard load configurations (Tang et al., 2025): shoulder-carrying, cradle-carrying, and hand-carrying. Finally, drawing on established firefighting training and competition practices, the 5 × 20-m shuttle sprint run was selected as the experimental test format. This task is a standard event in China’s firefighting and rescue competitions. It is designed to simulate the high-intensity, short-duration physical demands of specific rescue scenarios, such as emergency medical response or other time-critical operations where speed in transporting equipment is essential, rather than the sustained efforts characteristic of structural fire suppression.

### Experimental participants and sample size

The study employed a three-factor mixed experimental design. Fatigue state was treated as a within-subjects factor, meaning each participant completed tests under all five fatigue conditions. In contrast, task load and rescue method were between-subjects factors; for each test under a specific fatigue state, a participant was randomly assigned to only one combination of task load and rescue method. This approach balanced the need to collect comprehensive data with the practical constraint of preventing excessive fatigue accumulation.

The assignment protocol was designed as a cyclic Latin square to ensure data balance. Each firefighter completed five rescue task tests, corresponding to the five fatigue states, with a minimum of 48 h between tests to ensure recovery. One of the 9 task load–method combinations was randomly selected for each test. Thus, 9 participants across different fatigue states completed one full cycle of 45 sample groups (see Table 1). Across 63 participants (7 cycles), 315 valid samples were obtained, ensuring each sample category contained at least 7 data points.

**TABLE 1:** Cyclic participant assignment schedule for the mixed-experimental design (first of seven cycles shown).

Number of test rounds	Participant number	Fatigue states	Rescue task loads	Rescue methods
First round	1	Non-fatigue	10 kg (small load)	Shoulder-carrying
	2	Non-fatigue	20 kg (medium load)	Shoulder-carrying
	3	Non-fatigue	30 kg (large load)	Shoulder-carrying
	4	Non-fatigue	10 kg (small load)	Cradle-carrying
	5	Non-fatigue	20 kg (medium load)	Cradle-carrying
	6	Non-fatigue	30 kg (large load)	Cradle-carrying
	7	Non-fatigue	10 kg (small load)	Hand-carrying
	8	Non-fatigue	20 kg (medium load)	Hand-carrying
	9	Non-fatigue	30 kg (large load)	Hand-carrying
	1	Whole-body fatigue	20 kg (medium load)	Hand-carrying
	2	Whole-body fatigue	30 kg (large load)	Hand-carrying
	3	Whole-body fatigue	10 kg (small load)	Shoulder-carrying
	4	Whole-body fatigue	20 kg (medium load)	Shoulder-carrying
	5	Whole-body fatigue	30 kg (large load)	Shoulder-carrying
	6	Whole-body fatigue	10 kg (small load)	Cradle-carrying
	7	Whole-body fatigue	20 kg (medium load)	Cradle-carrying
	8	Whole-body fatigue	30 kg (large load)	Cradle-carrying
	9	Whole-body fatigue	10 kg (small load)	Hand-carrying
	1	Mild knee fatigue	30 kg (large load)	Cradle-carrying
	2	Mild knee fatigue	10 kg (small load)	Hand-carrying
	3	Mild knee fatigue	20 kg (medium load)	Hand-carrying
	4	Mild knee fatigue	30 kg (large load)	Hand-carrying
	5	Mild knee fatigue	10 kg (small load)	Shoulder-carrying
	6	Mild knee fatigue	20 kg (medium load)	Shoulder-carrying
	7	Mild knee fatigue	30 kg (large load)	Shoulder-carrying
	8	Mild knee fatigue	10 kg (small load)	Cradle-carrying
	9	Mild knee fatigue	20 kg (medium load)	Cradle-carrying
	1	Moderate knee fatigue	10 kg (small load)	Cradle-carrying
	2	Moderate knee fatigue	20 kg (medium load)	Cradle-carrying
	3	Moderate knee fatigue	30 kg (large load)	Cradle-carrying
	4	Moderate knee fatigue	10 kg (small load)	Hand-carrying
	5	Moderate knee fatigue	20 kg (medium load)	Hand-carrying
	6	Moderate knee fatigue	30 kg (large load)	Hand-carrying
	7	Moderate knee fatigue	10 kg (small load)	Shoulder-carrying
	8	Moderate knee fatigue	20 kg (medium load)	Shoulder-carrying
	9	Moderate knee fatigue	30 kg (large load)	Shoulder-carrying
	1	Severe knee fatigue	20 kg (medium load)	Shoulder-carrying
	2	Severe knee fatigue	30 kg (large load)	Shoulder-carrying
	3	Severe knee fatigue	10 kg (small load)	Shoulder-carrying
	4	Severe knee fatigue	20 kg (medium load)	Cradle-carrying
	5	Severe knee fatigue	30 kg (large load)	Cradle-carrying
	6	Severe knee fatigue	10 kg (small load)	Cradle-carrying
	7	Severe knee fatigue	20 kg (medium load)	Hand-carrying
	8	Severe knee fatigue	30 kg (large load)	Hand-carrying
	9	Severe knee fatigue	10 kg (small load)	Hand-carrying

This table illustrates the assignment of the nine participants in the first cycle to their unique combination of conditions for each fatigue state. Fatigue state is a within-subjects factor (each participant’s number appears in all five fatigue state blocks). At the same time, task load and rescue methods are between-subject factors within each fatigue state (each participant is assigned to only one load-method combination per state). This schedule was repeated over seven cycles (N = 63 participants) to ensure balanced data collection across all 45 experimental cells.

The sample size was determined *a priori* using G*Power software (Version 3.1). Based on a pilot study and previous related research on firefighter physiology (Tang et al., 2025), we estimated a medium-to-large effect size (f = 0.25). For a mixed-design ANOVA with within-between interaction, with an alpha level of 0.05 and a desired statistical power (1 - β) of 0.80, the analysis indicated a required total sample size of approximately 54 participants. Accordingly, this study recruited 63 professional, healthy male firefighters from the Guilin Fire and Rescue Detachment as experimental participants, with their baseline characteristics summarized in Table 2. To provide context for the physical fitness of the cohort, the mean Body Mass Index (BMI) was 25.2 ± 2.1 kg/m^2^. Additionally, their mean performance time for the 5 × 20-m shuttle sprint run in a non-fatigued state was 28.4 ± 2.1 s, and the mean time to exhaustion on the whole-body fatigue induction protocol was 18.5 ± 3.2 min. Descriptive statistics for task completion times and oxygen consumption values across all experimental conditions are provided in Supplementary Appendix Tables S1, S2, respectively. All participants met the following inclusion criteria: good overall physical health, standard anatomical structure and function, no history of medication use, and no injuries or illnesses within the previous 6 months. Written informed consent was obtained from each participant before testing. The study was conducted per the principles of the Declaration of Helsinki and received ethical approval from the Ethics Committee of Guangxi Normal University (Approval No. 20230419001).

**TABLE 2:** Participant basic information.

Number	Age (years)	Height (cm)	Weight (kg)	Years of service
63	29.24 ± 4.53	175.43 ± 9.86	77.68 ± 6.52	4.13 ± 1.67

### Experimental equipment

Whole-body fatigue was induced using a treadmill (Merach Phantom X1, China) and a Polar heart rate monitor (Polar H9, Finland). Localized knee joint fatigue was induced with the Humac Norm isokinetic dynamometer system (CSMI, United States). Oxygen consumption was measured using a wearable metabolic system (COSMED K5, Italy). A multifunctional fitness training sandbag (KR Fitness, China) was employed to simulate the weight of standard rescue equipment, while marker cones were used to delineate the positions for shuttle runs.

### Experimental procedure

**FIGURE 1:**
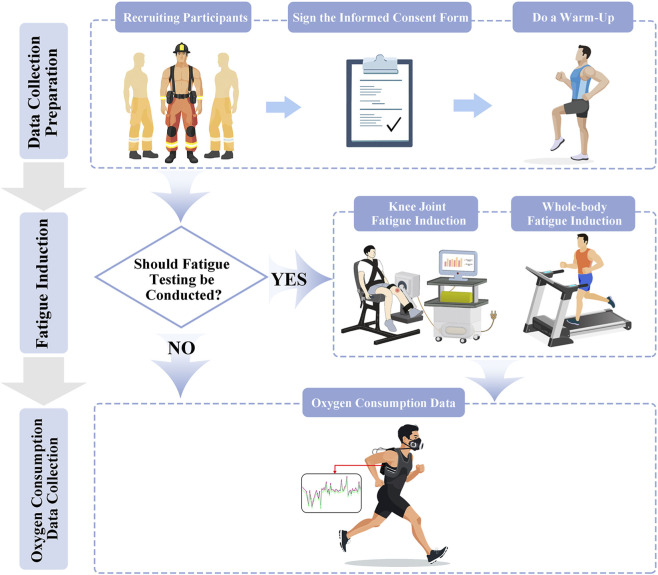
Test flowchart.

This experimental procedure consists of three parts, as detailed in Figure 1 below.

#### Data collection preparation

Before the experiment, the wearable metabolic system was preheated and calibrated according to the user manual, including turbine, desiccant, standard gas, air, and respiratory delay calibration. Upon arrival, participants signed informed consent forms and registered basic demographic information, including height, weight, age, and years of service. The laboratory temperature was maintained at 22 °C–26 °C, and participants completed warm-up activities consisting of a 5-min jog followed by 5 min of hamstring and quadriceps stretching (each stretch held for 30 s with 20-s intervals, repeated for three sets). Before the formal test commenced, all non-essential personnel exited the testing area, and indoor air circulation was ensured. To control for potential confounders, all participants were instructed to maintain their regular sleep patterns, refrain from strenuous exercise, alcohol, and caffeine consumption for 24 h before each testing session, and arrive at the laboratory in a euhydrated state.

#### Fatigue induction

After data acquisition preparation, participants proceeded directly to testing if assigned to the non-fatigued condition. For fatigued conditions, induction followed one of two established models:

1. Whole-Body Fatigue: Per standard graded exercise testing protocols for inducing volitional exhaustion in healthy adults (Armstrong et al., 2026), participants began running at 5 km/h with a 0% incline. Speed was maintained for 14 min, while the incline increased by 2% every 2 min until reaching 14%. Thereafter, incline remained constant, and speed increased by 0.5 km/h per minute. Following Zhang Xini et al. (Zhang et al., 2017), fatigue was achieved when participants reached 90% of their age-predicted maximum heart rate (220-age) and could no longer sustain the prescribed running speed.
2. Knee Joint Fatigue: Following established protocols (Gear, 2011), fatigue was induced bilaterally using an isokinetic dynamometer system. Participants performed the procedure sequentially for both legs. They were seated with hips, ankles, and torso securely fixed, and gravity correction was applied to eliminate equipment weight effects. The induction procedure consisted of 3 trial knee flexion/extension cycles at an angular velocity of 60°/s, followed by a 1-min rest. Participants then performed 5 maximal knee extension repetitions at the same angular velocity, with peak moment recorded. After a 40-s rest, they executed repeated extensions at 60°/s until fatigue. Fatigue severity was classified as mild, moderate, or severe when the maximum knee extension moment dropped below 75%, 50%, or 25% of peak value, respectively, for three consecutive trials (Gear, 2011).

#### Oxygen consumption data collection

**FIGURE 2:**
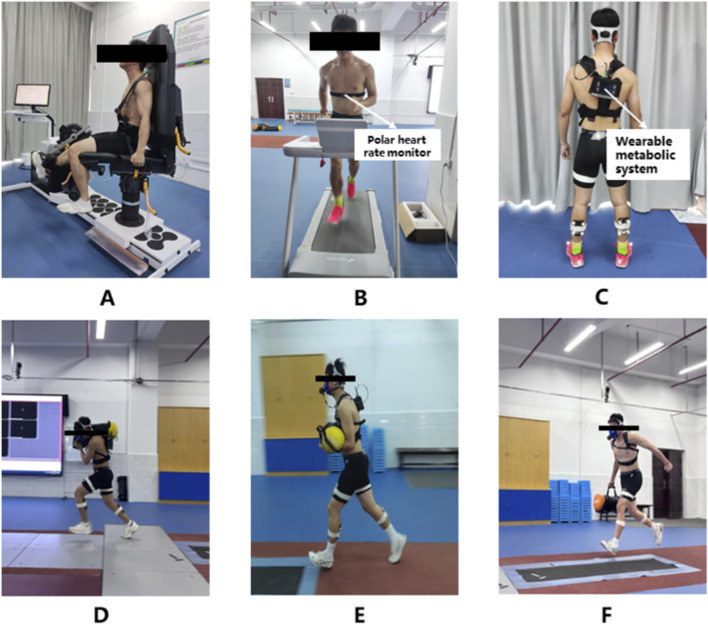
Experimental Process Diagram. **(A)** Knee fatigue induction; **(B)** Whole-body fatigue induction; **(C)** Instrument wear diagram; **(D)** Shoulder-carrying data collection diagram; **(E)** Cradle-carrying data collection diagram; **(F)** Hand-carrying data collection diagram.

The wearable metabolic system was secured to each participant’s back. During the experiment, 63 firefighters randomly completed five tests according to the task schedule (Figure 2), with a minimum interval of 48 h between tests to prevent residual fatigue effects from influencing subsequent trials. This recovery period is well-established in exercise physiology literature for ensuring complete neuromuscular and metabolic recovery following high-intensity fatigue protocols, and was confirmed in our pilot testing to be sufficient (Refalo et al., 2023). A 20-m straight shuttle sprint track was delineated in the laboratory using marker cones. Participants were instructed to complete five repetitions of the 20-m shuttle sprint at maximum speed for five back-and-forth cycles (5 × 20-m). Raw peak oxygen consumption data were recorded in real time using the Omnia software integrated with the wearable metabolic system.

### Data processing and statistical analysis

Raw maximum oxygen consumption data were processed using the Omnia software integrated with the wearable metabolic system. This study primarily examined the intrinsic relationships among firefighters’ fatigue states, rescue task loads, and rescue methods. However, oxygen consumption is also influenced by body weight and task load magnitude (Tang et al., 2025). Data were standardized relative to body weight and task load to isolate better the oxygen consumption responses specific to the task execution. The standardized oxygen consumption metric was calculated as follows:

Standardized Oxygen Consumption (mL/min/kg^2^) = VO_2_max (mL/min) ÷ Body Weight (kg) ÷ Task Load (kg).

All statistical analyses were performed using SPSS 26.0 software (IBM, NY, United States). A mixed-effects analysis of variance (ANOVA) was employed. The model included ‘Participant’ as a random effect to account for inter-individual variability due to the repeated measures on fatigue states. Fatigue states, task loads, and rescue methods were entered as fixed effects, and the covariance structure was determined based on model fit indices (AIC and BIC). For all significant effects, the F-statistic, p-value, and partial eta-squared ( $\left.{ƞ}_{p}^{2}\right)$ as a measure of effect size was reported. Post-hoc pairwise comparisons for significant interactions were conducted and adjusted for multiple comparisons using the Bonferroni correction. Statistical significance was set at P < 0.05.

## Results

### Results of analysis of variance

As shown in Table 3, the main effects of fatigue states and rescue methods on standardized oxygen consumption and the interactions between fatigue states and rescue methods and between fatigue states and rescue task loads were not significant (P > 0.05). However, the main effect of rescue task loads, the interaction between rescue task loads and rescue methods, and the interactions between fatigue states, rescue task loads, and rescue methods were significant (P < 0.05).

**TABLE 3:** Summary of ANOVA results.

Independent variables	F ratio	P-value	${ƞ}_{p}^{2}$
Fatigue states	1.620	0.169	0.020
Rescue task loads	620.606	<0.001	0.798
Rescue methods	1.027	0.359	0.006
Fatigue states × rescue task loads	0.698	0.693	0.017
Fatigue states × rescue methods	0.327	0.955	0.008
Rescue task loads × rescue methods	2.832	0.025	0.035
Fatigue states × rescue task loads × rescue methods	2.018	0.012	0.093

${ƞ}_{p}^{2}$ was used to report effect sizes, specifying 0.01–0.06 as a small effect size, 0.06–0.14 as a medium effect size, and greater than 0.14 as a large effect size.

### Multiple comparisons results for main effects

As shown in Table 4, the standardized oxygen consumption for the medium- and large-load shuttle sprint run tasks was lower than that for the small-load task (P < 0.05). Furthermore, the standardized oxygen consumption for the large-load shuttle sprint run task was lower than that for the medium-load task (P < 0.05).

**TABLE 4:** Multiple Comparison Results of the Main Effect in Rescue Task Loads (N = 63, unit: mL/min/kg^2^).

Rescue task loads	Standardized oxygen consumption
10 kg (small load)	4.718 ± 1.043d
20 kg (medium load)	2.464 ± 0.616e
30 kg (large load)	1.615 ± 0.317f

Identical letters indicate differences are not statistically significant (P > 0.05), while different letters indicate statistically significant differences (P < 0.05).

### Multiple Comparison Results for Interaction Effects

**FIGURE 3:**
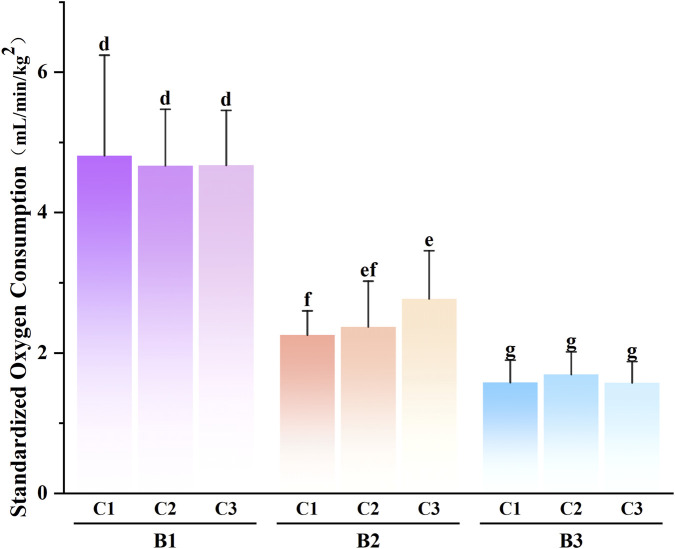
Multiple Comparison Results of the Interaction Effect between Rescue Task Loads and Rescue Methods. (B1) 10 kg (small load); (B2) 20 kg (medium load); (B3) 30 kg (large load); (C1) Shoulder-carrying; (C2) Cradle-carrying; (C3) Hand-carrying. Identical letters indicate differences are not statistically significant (P > 0.05), while different letters indicate statistically significant differences (P < 0.05). The sample size for each experimental condition is n = 7.

Regarding the interaction between rescue task loads and rescue methods (Figure 3), *post hoc* tests revealed that the standardized oxygen consumption for performing medium- and large-load shuttle sprint runs using any rescue method was lower than that for performing small-load tasks (P < 0.05). The standardized oxygen consumption for performing a large-load shuttle sprint run using any rescue method was lower than that for performing medium-load tasks (P < 0.05).

**FIGURE 4:**
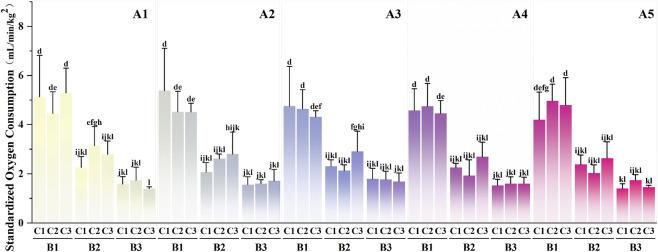
Multiple Comparison Results of the Interaction Effects between Fatigue States, Rescue Task Loads, and Rescue Methods. (A1) Non-fatigue; (A2) Whole-body fatigue; (A3) Mild knee fatigue; (A4) Moderate knee fatigue; (A5) Severe knee fatigue; (B1) 10 kg (small load); (B2) 20 kg (medium load); (B3) 30 kg (large load); (C1) Shoulder-carrying; (C2) Cradle-carrying; (C3) Hand-carrying. Identical letters indicate differences are not statistically significant (P > 0.05), while different letters indicate statistically significant differences (P < 0.05). The sample size for each experimental condition is n = 7.

Post-hoc analysis of the interaction among fatigue states, rescue task loads, and rescue methods (Figure 4) revealed several notable patterns organized thematically below for clarity.

First, standardized oxygen consumption during medium- and large-load shuttle sprint tasks, regardless of rescue method or fatigue condition, was consistently lower than that observed under the following small-load conditions: (i) shoulder- or hand-carrying under non-fatigue; (ii) shoulder-carrying under whole-body fatigue; (iii) shoulder- or cradle-carrying under mild and moderate knee fatigue; and (iv) cradle- or hand-carrying under severe knee fatigue (P < 0.05).

Second, standardized oxygen consumption during medium-load tasks using shoulder- or hand-carrying under non-fatigue, large-load tasks using any rescue method under non-fatigue, and medium- and large-load tasks using any rescue method under whole-body or knee fatigue was lower than that during small-load conditions involving (i) cradle-carrying under non-fatigue; (ii) cradle- or hand-carrying under whole-body fatigue; and (iii) hand-carrying under moderate knee fatigue (P < 0.05).

Third, standardized oxygen consumption during medium-load tasks with shoulder- or hand-carrying under non-fatigue, large-load tasks with any method under non-fatigue, medium- and large-load tasks with any method under whole-body fatigue, small-load tasks with shoulder- or cradle-carrying under mild knee fatigue, large-load tasks with any method under mild knee fatigue, and medium- and large-load tasks with any method under moderate and severe knee fatigue was lower than that during small-load conditions involving (i) hand-carrying under mild knee fatigue and (ii) shoulder-carrying under severe knee fatigue (P < 0.05).

Additionally, small-load tasks performed using cradle-carrying under non-fatigue elicited higher standardized oxygen consumption compared with medium-load tasks performed using shoulder- or hand-carrying under non-fatigue, large-load tasks with any method under non-fatigue, medium-load tasks with shoulder- or cradle-carrying under whole-body or mild knee fatigue, large-load tasks with any method under whole-body or mild knee fatigue, and medium- and large-load tasks with any method under moderate and severe knee fatigue (P < 0.05).

Furthermore, medium-load tasks performed using hand-carrying under mild knee fatigue elicited higher standardized oxygen consumption compared with large-load tasks performed using any method under non-fatigue, whole-body fatigue, or moderate knee fatigue, as well as large-load tasks with hand-carrying under mild knee fatigue or with shoulder- and hand-carrying under severe knee fatigue (P < 0.05).

Finally, large-load tasks performed using hand-carrying under non-fatigue resulted in lower standardized oxygen consumption than medium-load tasks under whole-body fatigue (P < 0.05).

## Discussion

### Independent effects of fatigue states, rescue task loads, and rescue methods on oxygen consumption

The results of this study indicate that firefighters did not show significant differences in oxygen consumption across varying fatigue states during rescue tasks. This finding contrasts with previous studies, emphasizing that body fatigue significantly limits task performance and substantially increases oxygen consumption (Ras et al., 2024; Tang et al., 2025; Behrens et al., 2023). Fatigue does not completely suppress physiological function but instead activates compensatory mechanisms that help sustain performance (Purto et al., 2024; Arya et al., 2021). For example, other muscle groups can compensate for reduced strength due to localized muscle fatigue by increasing recruitment and synergistic action (Denby et al., 2020). Moreover, the impact of fatigue on performance is influenced by the characteristics of the study population (Behrens et al., 2023). The firefighters in this study, with a mean BMI of 25.2 kg/m^2^ and a non-fatigued shuttle run time of 28.4 s, represent a professional group required to perform high-intensity tasks. While their BMI is comparable to or higher than the general population’s, their ability to maintain stable oxygen consumption during demanding rescue tasks suggests a task-specific fitness and metabolic adaptation that may not be reflected in BMI alone. This reflects a functional adaptability to their occupational demands. As a result, even under fatigued conditions, firefighters sustain relatively stable oxygen consumption, reflecting strong adaptability and physiological resilience. These findings suggest that fatigue is not the sole determinant of oxygen consumption in firefighters. The physiological basis for this stable performance across fatigue states warrants further investigation.

This study found no significant differences in oxygen consumption across different rescue methods. This finding differs from those of Von et al. (2006), who reported differences in oxygen consumption among specific dragging strategies. This discrepancy may stem from fundamental differences in the task modes: Von et al. (2006) investigated a continuous dragging task, whose biomechanical and energetic demands are likely to differ significantly from the high-intensity, multi-round shuttle sprint run involving frequent directional changes used in our study. During shuttle sprints, firefighters can dynamically adjust their posture and force application with each turn, potentially offsetting theoretical biomechanical disadvantages of specific carrying methods. This suggests that the physiological impact of a rescue method may be highly task-specific, and findings from one task modality cannot be directly extrapolated to another.

A notable result of this study is that performing large-load tasks demonstrated superior oxygen utilization efficiency. Prior research has established that load magnitude significantly affects oxygen consumption (Tang et al., 2025; Mackie et al., 2005). Loads exceeding 10% of body weight increase respiratory rate and depth, thereby elevating oxygen expenditure (Epstein et al., 1988; Li et al., 2003; Quesada et al., 2000). In emergency rescue operations, heavier loads increase exertion intensity, leading to higher heart rate, ventilation volume, and oxygen consumption, shortening the operational time of breathing apparatus and posing safety risks (Tang et al., 2025). However, previous studies largely measured oxygen consumption by calculating air cylinder depletion, i.e., subtracting the residual oxygen volume after exercise from the initial oxygen supply (Kesler et al., 2018b). In contrast, this study employed maximum oxygen consumption during the task, standardized relative to load size, providing a more accurate indicator of oxygen utilization efficiency per unit of task weight. This methodological difference likely explains the observed oxygen utilization advantage in large-load tasks. Furthermore, real-world rescue operations often involve multiple-return movement patterns, whereas prior studies focused on single, long-duration tasks (Epstein et al., 1988; Li et al., 2003; Quesada et al., 2000). Under a multiple-return framework, small-load tasks require more trips to complete the same task volume, whereas large-load tasks require fewer trips. Although oxygen consumption per trip is lower with small loads, the cumulative oxygen cost across multiple trips may not decrease. Integrating previous and current findings suggests that large-load tasks are more oxygen-efficient for short-duration, multiple-return operations. In contrast, small-load tasks may be better suited to long-duration, single-return scenarios. Future studies should refine task classifications and load categories to develop more targeted optimization strategies for firefighting rescue operations.

### Interactive effects of fatigue states, rescue task loads, and rescue methods on oxygen consumption

This study demonstrates that firefighters achieve superior oxygen utilization efficiency during large-load tasks, regardless of fatigue status or rescue method. Physically, fatigue imposes greater demands on muscular strength, cardiopulmonary function, and neuromuscular coordination (Rapti et al., 2023; Vasconcelos and Sousa, 2024), particularly under the high-intensity conditions created by large-load tasks. To sustain performance in these scenarios, the body must actively regulate metabolic homeostasis (Rapti et al., 2023), thereby enhancing oxygen consumption efficiency. This finding supports and allows us to elaborate on the “exercise compensation mechanisms under fatigue conditions” theory proposed in prior research (Rapti et al., 2023; Vasconcelos and Sousa, 2024). This theory posits that as the body becomes fatigued, it does not simply experience a linear decline in function. Instead, it activates a suite of compensatory adaptations to maintain performance and metabolic homeostasis for as long as possible. These adaptations can be observed in specific physiological mechanisms. Neuromuscular compensation may occur as the nervous system increases the recruitment of additional motor units to counteract force loss (Behrens et al., 2023; Denby et al., 2020). Conversely, metabolic regulation may involve a shift in substrate utilization to preserve performance under metabolic stress (Rapti et al., 2023). In the context of our study, the firefighters’ maintained oxygen efficiency during large-load tasks under fatigue likely reflects such compensatory regulation. Their extensive training likely enhances this capacity, enabling them to leverage these mechanisms effectively to sustain performance when untrained individuals might experience a significant decline. Thus, the superior oxygen utilization we observed during large-load tasks is not merely despite fatigue but may be facilitated by activating these very compensation mechanisms. By contrast, medium- and small-load tasks under fatigue conditions fail to provide sufficient stimulus to fully activate latent metabolic regulatory mechanisms, thereby exhibiting a “small load–low efficiency” oxygen consumption pattern.

Regarding rescue methodologies, medium- and small-load tasks rely more heavily on smaller muscle groups to maintain movement stability. Such reliance necessitates frequent postural adjustments and fine motor control, increasing energy expenditure and oxygen consumption (Tang et al., 2025). In contrast, large-load tasks predominantly recruit larger muscle groups, which generate greater mechanical output per unit of contraction. Because these groups are primarily engaged in overcoming gravitational loads, they achieve higher oxygen consumption efficiency than the sustained, low-intensity contractions required of smaller muscle groups (Muscolino, 2022; Winter 2009).

In addition, the shuttle sprint run employed in this study relies predominantly on anaerobic metabolism. Large-load tasks induce a stronger anaerobic metabolic response, whereas medium- and small-load tasks, being of lower overall intensity, involve a relatively greater contribution of aerobic metabolism. This greater reliance on aerobic metabolism increases oxygen consumption, providing another explanation for their lower oxygen utilization efficiency.

From a practical perspective, large-load tasks often involve high-risk, high-priority scenarios that elicit heightened psychological mobilization and attentional focus. Under the combined influence of psychological arousal and physiological activation, firefighters can more effectively mobilize resources, maintain metabolic regulation, and exhibit strong task adaptability—even under fatigue or when employing different rescue methods. Future research should seek to validate the generalizability of these findings by varying exercise formats and further examining task efficiency, including indices such as energy-to-work conversion efficiency.

### Clarification on standardized oxygen consumption and practical relevance

The present study employed a standardized oxygen consumption metric (mL/min/kg^2^) to evaluate the efficiency of oxygen utilization per unit of load carried, rather than the absolute physiological cost. We acknowledge that absolute oxygen consumption (L/min) unequivocally increases with greater task loads, leading to higher ventilatory rates and accelerated self-contained breathing apparatus (SCBA) depletion, as robustly demonstrated in previous literature (Tang et al., 2025; Epstein et al., 1988; Li et al., 2003; Quesada et al., 2000). This relationship is a fundamental and critical consideration for operational safety. However, our focus was on a different operational question: in scenarios requiring multiple shuttles to move a given total mass of equipment, which strategy minimizes the oxygen cost per kilogram transported? Our findings of lower standardized consumption for large-load tasks suggest that, in a multi-trip framework, completing the total work with fewer trips and heavier loads may be more efficient in terms of oxygen utilization than more frequent trips with lighter loads. This implies a potential trade-off between a single heavy lift’s high absolute metabolic cost and the improved efficiency and reduced time achievable at the task level. Therefore, the conclusion that high-load tasks optimize oxygen consumption efficiency is framed within this specific context of short-duration, multi-trip logistics and should be interpreted as a strategy to enhance overall task completion efficiency under time and oxygen constraints, rather than to reduce the instantaneous metabolic rate.

### Limitations and future perspectives

This study has several limitations that should be considered when interpreting the findings. First, the physiological explanations proposed herein, particularly regarding compensatory mechanisms under fatigue, remain speculative as we did not measure neuromuscular activity, central command, or metabolic markers. Future research incorporating electromyography (EMG) and blood biomarkers would be valuable in elucidating the underlying mechanisms. Second, this study employed a standardized oxygen consumption (mL/min/kg^2^) as its primary dependent variable. This is an unconventional approach in exercise physiology, where reporting absolute (L/min) and body mass-relative (mL·kg^−1^·min^−1^) oxygen consumption is standard. The specific research aim drove our deliberate choice: to compare the efficiency of oxygen utilization across different task loads rather than merely the gross physiological cost. While standard metrics would unequivocally show higher oxygen consumption with larger loads, our chosen metric provides a normalized measure of the oxygen cost per kilogram of load, which we argue is a more operationally relevant efficiency metric for firefighters who must move equipment of varying weights. Future studies could benefit from reporting standard physiological and task-specific efficiency metrics to provide a more comprehensive picture. Third, all assessments were conducted without firefighters wearing their complete personal protective equipment (PPE) and SCBA. Adding PPE and SCBA would substantially increase the absolute metabolic and biomechanical demands (Kesler et al., 2018b; Kesler et al., 2018c; Morel et al., 2014), potentially altering the efficiency relationships observed here. Therefore, the direct translation of our specific efficiency metrics to SCBA duration in structural firefighting is limited. Our results most apply to similar sprint-based rescue tasks in environments where PPE/SCBA is not required or is minimal, or to the logistical planning of equipment movement in support roles. Future research incorporating full PPE and direct SCBA air consumption measurements is crucial to validate the practical implications of these efficiency findings for firefighter safety and operational capacity. Finally, the experimental tasks simulated a specific type of short-duration, high-intensity, multi-trip load carriage. Therefore, generalizing these findings to the entire spectrum of firefighting activities (e.g., prolonged operations, technical rescues) is not warranted. Our results most directly apply to similar sprint-based rescue tasks in environments where PPE is not required or is minimal.

## Conclusions

All fatigue states and rescue methods can be applied without distinction to firefighting rescue tasks, with large-load tasks associated with a lower oxygen cost per unit of load carried. (Bai et al., 2021). The findings suggest that prioritizing large-load tasks under laboratory, time-sensitive, or oxygen-limited conditions may improve overall rescue resource utilization, highlighting a “small load–low operational efficiency” phenomenon in the context of multi-trip shuttle runs, pending field validation. Therefore, it is recommended that firefighters prioritize large-load tasks (30 kg) under time-sensitive or oxygen-limited emergency conditions to improve overall rescue efficiency and optimize resource utilization.

## Data Availability

The data analyzed in this study is subject to the following licenses/restrictions: The datasets used and/or analyzed during the current study available from the corresponding author on reasonable request. Requests to access these datasets should be directed to liuwg@mailbox.gxnu.edu.cn.
