# Supplementary material for: Oxygen consumption efficiency in firefighters: roles of fatigue and rescue task
Source: Front Physiol. 2025 Nov 26;16:1708050. doi: 10.3389/fphys.2025.1708050 (PMC12689312; doi:10.3389/fphys.2025.1708050)
Supplement: Supplementary file 1 [file Table1.docx]

# Appendix

**Appendix Table S1. Descriptive Statistics for Task Completion Time (seconds) Across All Experimental Conditions.**

| **Fatigue State** | **Task Load (kg)** | **Rescue Method** | **Mean ± SD** | **Median** |
| --- | --- | --- | --- | --- |
| Non-Fatigue | 10 | Shoulder | 23.89±0.96 | 23.75 |
| Non-Fatigue | 10 | Hand | 24.00±1.50 | 24.00 |
| Non-Fatigue | 10 | Cradle | 22.88±0.76 | 22.88 |
| Non-Fatigue | 20 | Shoulder | 24.34±1.07 | 24.34 |
| Non-Fatigue | 20 | Hand | 27.34±1.87 | 27.34 |
| Non-Fatigue | 20 | Cradle | 23.58±0.91 | 23.58 |
| Non-Fatigue | 30 | Shoulder | 26.84±1.23 | 26.84 |
| Non-Fatigue | 30 | Hand | 35.46±5.67 | 35.46 |
| Non-Fatigue | 30 | Cradle | 26.79±1.45 | 26.79 |
| Whole body Fatigue | 10 | Shoulder | 24.07±1.32 | 24.07 |
| Whole body Fatigue | 10 | Hand | 24.94±1.62 | 24.94 |
| Whole body Fatigue | 10 | Cradle | 23.38±0.89 | 23.38 |
| Whole body Fatigue | 20 | Shoulder | 25.14±1.24 | 25.14 |
| Whole body Fatigue | 20 | Hand | 29.00±2.34 | 29.00 |
| Whole body Fatigue | 20 | Cradle | 24.48±1.02 | 24.48 |
| Whole body Fatigue | 30 | Shoulder | 26.89±1.38 | 26.89 |
| Whole body Fatigue | 30 | Hand | 38.76±7.89 | 38.76 |
| Whole body Fatigue | 30 | Cradle | 29.51±2.34 | 29.51 |
| Mild knee fatigue | 10 | Shoulder | 23.68±1.12 | 23.68 |
| Mild knee fatigue | 10 | Hand | 24.76±1.45 | 24.76 |
| Mild knee fatigue | 10 | Cradle | 23.51±0.98 | 23.51 |
| Mild knee fatigue | 20 | Shoulder | 24.98±1.23 | 24.98 |
| Mild knee fatigue | 20 | Hand | 27.14±1.67 | 27.14 |
| Mild knee fatigue | 20 | Cradle | 26.48±1.45 | 26.48 |
| Mild knee fatigue | 30 | Shoulder | 26.89±1.56 | 26.89 |
| Mild knee fatigue | 30 | Hand | 35.67±6.45 | 35.67 |
| Mild knee fatigue | 30 | Cradle | 27.34±1.78 | 27.34 |
| Moderate knee fatigue | 10 | Shoulder | 23.89±1.07 | 23.89 |
| Moderate knee fatigue | 10 | Hand | 23.76±1.23 | 23.76 |
| Moderate knee fatigue | 10 | Cradle | 23.68±1.12 | 23.68 |
| Moderate knee fatigue | 20 | Shoulder | 25.34±1.45 | 25.34 |
| Moderate knee fatigue | 20 | Hand | 29.12±2.34 | 29.12 |
| Moderate knee fatigue | 20 | Cradle | 26.78±1.67 | 26.78 |
| Moderate knee fatigue | 30 | Shoulder | 28.12±1.89 | 28.12 |
| Moderate knee fatigue | 30 | Hand | 38.45±7.23 | 38.45 |
| Moderate knee fatigue | 30 | Cradle | 28.34±2.01 | 28.34 |
| Severe Knee Fatigue | 10 | Shoulder | 24.12±1.34 | 24.12 |
| Severe Knee Fatigue | 10 | Hand | 24.34±1.56 | 24.34 |
| Severe Knee Fatigue | 10 | Cradle | 23.89±1.23 | 23.89 |
| Severe Knee Fatigue | 20 | Shoulder | 25.67±1.45 | 25.67 |
| Severe Knee Fatigue | 20 | Hand | 27.89±2.01 | 27.89 |
| Severe Knee Fatigue | 20 | Cradle | 25.12±1.34 | 25.12 |
| Severe Knee Fatigue | 30 | Shoulder | 28.34±1.67 | 28.34 |
| Severe Knee Fatigue | 30 | Hand | 38.12±8.45 | 38.12 |
| Severe Knee Fatigue | 30 | Cradle | 27.89±2.12 | 27.89 |

**Appendix Table S2. Descriptive Statistics for Oxygen Consumption Across All Experimental Conditions.**

| **Fatigue State** | **Task Load (kg)** | **Rescue Method** | **VO₂ (ml/kg/min), Mean ± SD** | **VO₂ (L/min), Mean ± SD** |
| --- | --- | --- | --- | --- |
| Non-Fatigue | 10 | Shoulder | 29.63±4.72 | 4.12±1.31 |
| Non-Fatigue | 10 | Hand | 31.84±3.15 | 3.97±0.78 |
| Non-Fatigue | 10 | Cradle | 30.18±5.12 | 3.29±0.47 |
| Non-Fatigue | 20 | Shoulder | 29.72±4.56 | 3.79±0.98 |
| Non-Fatigue | 20 | Hand | 33.53±6.23 | 3.98±0.67 |
| Non-Fatigue | 20 | Cradle | 32.87±6.45 | 3.87±1.23 |
| Non-Fatigue | 30 | Shoulder | 29.52±4.89 | 3.41±0.67 |
| Non-Fatigue | 30 | Hand | 32.25±4.78 | 3.56±0.45 |
| Non-Fatigue | 30 | Cradle | 31.38±3.67 | 3.61±0.89 |
| Whole body Fatigue | 10 | Shoulder | 32.67±5.23 | 4.56±1.45 |
| Whole body Fatigue | 10 | Hand | 32.12±3.89 | 3.56±0.67 |
| Whole body Fatigue | 10 | Cradle | 29.24±3.45 | 3.29±0.34 |
| Whole body Fatigue | 20 | Shoulder | 29.01±3.78 | 3.45±0.56 |
| Whole body Fatigue | 20 | Hand | 34.89±5.67 | 4.23±1.23 |
| Whole body Fatigue | 20 | Cradle | 33.04±4.56 | 3.89±0.78 |
| Whole body Fatigue | 30 | Shoulder | 30.26±4.12 | 3.34±0.45 |
| Whole body Fatigue | 30 | Hand | 34.56±5.89 | 3.98±0.89 |
| Whole body Fatigue | 30 | Cradle | 35.12±4.78 | 3.67±0.56 |
| Mild knee fatigue | 10 | Shoulder | 25.67±10.23 | 3.01±1.78 |
| Mild knee fatigue | 10 | Hand | 28.56±3.45 | 3.34±0.45 |
| Mild knee fatigue | 10 | Cradle | 22.34±8.67 | 2.45±1.23 |
| Mild knee fatigue | 20 | Shoulder | 31.23±3.89 | 3.34±0.34 |
| Mild knee fatigue | 20 | Hand | 33.45±5.12 | 4.12±1.45 |
| Mild knee fatigue | 20 | Cradle | 27.89±3.67 | 3.23±0.56 |
| Mild knee fatigue | 30 | Shoulder | 31.45±5.78 | 4.01±1.12 |
| Mild knee fatigue | 30 | Hand | 32.78±6.45 | 4.23±1.34 |
| Mild knee fatigue | 30 | Cradle | 33.67±4.89 | 3.78±0.78 |
| Moderate knee fatigue | 10 | Shoulder | 28.45±6.78 | 3.56±0.89 |
| Moderate knee fatigue | 10 | Hand | 28.34±4.56 | 3.67±0.67 |
| Moderate knee fatigue | 10 | Cradle | 29.78±5.23 | 3.45±0.78 |
| Moderate knee fatigue | 20 | Shoulder | 30.12±3.45 | 3.34±0.45 |
| Moderate knee fatigue | 20 | Hand | 32.67±4.78 | 3.89±0.89 |
| Moderate knee fatigue | 20 | Cradle | 28.45±7.89 | 3.12±1.23 |
| Moderate knee fatigue | 30 | Shoulder | 29.78±4.12 | 3.56±0.56 |
| Moderate knee fatigue | 30 | Hand | 34.12±5.67 | 3.78±0.67 |
| Moderate knee fatigue | 30 | Cradle | 31.45±4.89 | 3.45±0.45 |
| Severe Knee Fatigue | 10 | Shoulder | 24.78±7.89 | 3.23±1.34 |
| Severe Knee Fatigue | 10 | Hand | 28.34±5.12 | 3.45±0.78 |
| Severe Knee Fatigue | 10 | Cradle | 30.12±6.45 | 3.56±0.89 |
| Severe Knee Fatigue | 20 | Shoulder | 29.45±5.78 | 3.34±0.67 |
| Severe Knee Fatigue | 20 | Hand | 31.78±4.12 | 4.01±1.12 |
| Severe Knee Fatigue | 20 | Cradle | 27.12±3.89 | 3.23±0.56 |
| Severe Knee Fatigue | 30 | Shoulder | 29.67±4.56 | 3.45±0.45 |
| Severe Knee Fatigue | 30 | Hand | 33.45±6.78 | 3.89±0.78 |
| Severe Knee Fatigue | 30 | Cradle | 30.78±5.23 | 3.67±0.67 |
